# Supplementary material for: Coexistence of Multiple Endemic and Pandemic Lineages of the Rice Blast Pathogen
Source: mBio. 2018 Apr 3;9(2):e01806-17. doi: 10.1128/mBio.01806-17 (PMC5885030; doi:10.1128/mBio.01806-17)
Supplement: TABLE S2 [file mbo002183809st2.docx]

Table S2. Absolute divergence (dxy) per base pair between *Magnaporthe oryzae* lineages.

| Lineage | **2** | **3** | **4** | **5** | **6** |
| --- | --- | --- | --- | --- | --- |
| **1** | 0.00026 | 0.00026 | 0.00025 | 0.00028 | 0.00070 |
| **2** |  | 0.00024 | 0.00024 | 0.00027 | 0.00038 |
| **3** |  |  | 0.00023 | 0.00026 | 0.00048 |
| **4** |  |  |  | 0.00027 | 0.00042 |
| **5** |  |  |  |  | 0.00048 |

Values averaged across non-overlapping 100kb windows.
