# Supplementary material for: Coexistence of Multiple Endemic and Pandemic Lineages of the Rice Blast Pathogen
Source: mBio. 2018 Apr 3;9(2):e01806-17. doi: 10.1128/mBio.01806-17 (PMC5885030; doi:10.1128/mBio.01806-17)
Supplement: FIG S1 [file mbo002183809sf1.docx]

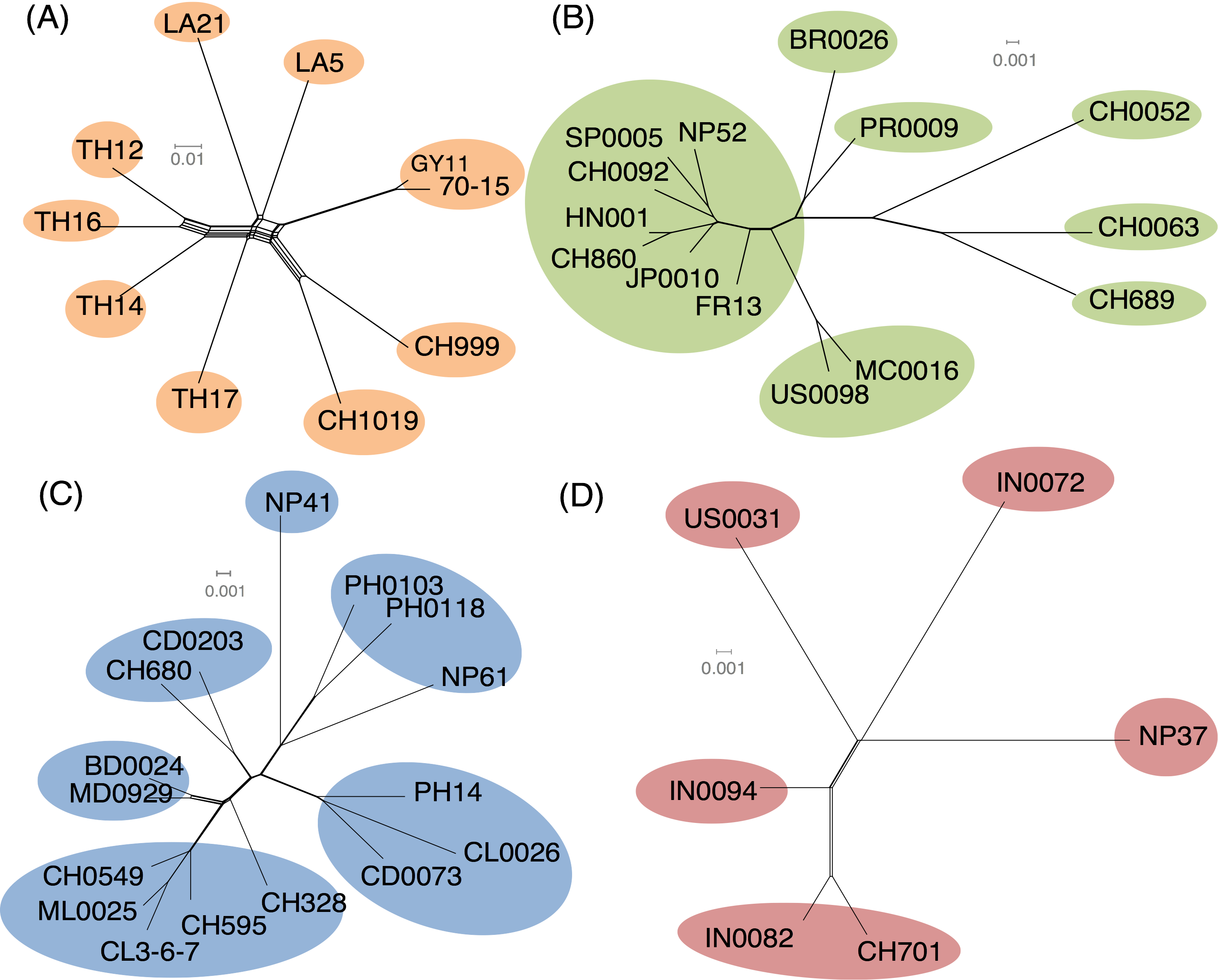


Figure S1. Neighbor-Net networks showing relationships between haplotypes identified on the basis of the full set of 16,370 SNPs without missing data, (A) in lineage 1, (B) in lineage 2, (C) in lineage 3, (D) in lineage 4.
