# Supplementary material for: Coexistence of Multiple Endemic and Pandemic Lineages of the Rice Blast Pathogen
Source: mBio. 2018 Apr 3;9(2):e01806-17. doi: 10.1128/mBio.01806-17 (PMC5885030; doi:10.1128/mBio.01806-17)
Supplement: FIG S2 [file mbo002183809sf2.docx]

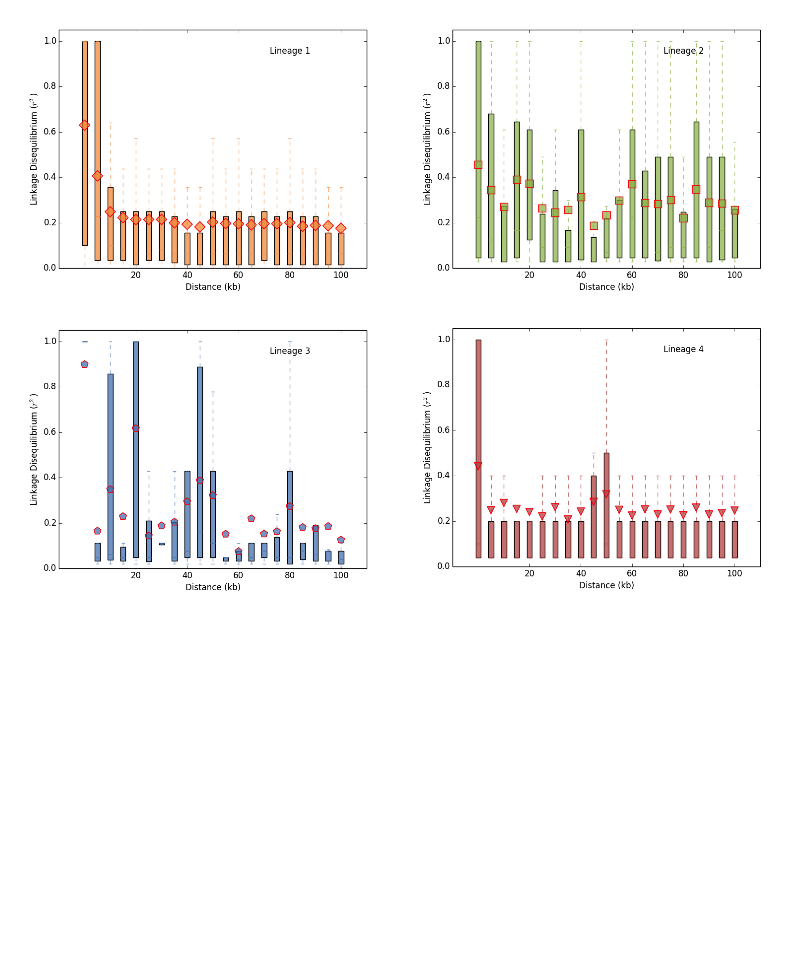


Figure S2. Boxplots showing linkage disequilibrium (r^2^) values for classes of SNP pairwise distances. Only SNPs distant by less than 100 kb and with a minor allele frequency of more than 10% are shown. Red markers represent averaged values.
