# Supplementary material for: Coexistence of Multiple Endemic and Pandemic Lineages of the Rice Blast Pathogen
Source: mBio. 2018 Apr 3;9(2):e01806-17. doi: 10.1128/mBio.01806-17 (PMC5885030; doi:10.1128/mBio.01806-17)
Supplement: FIG S3 [file mbo002183809sf3.pdf]

(A)

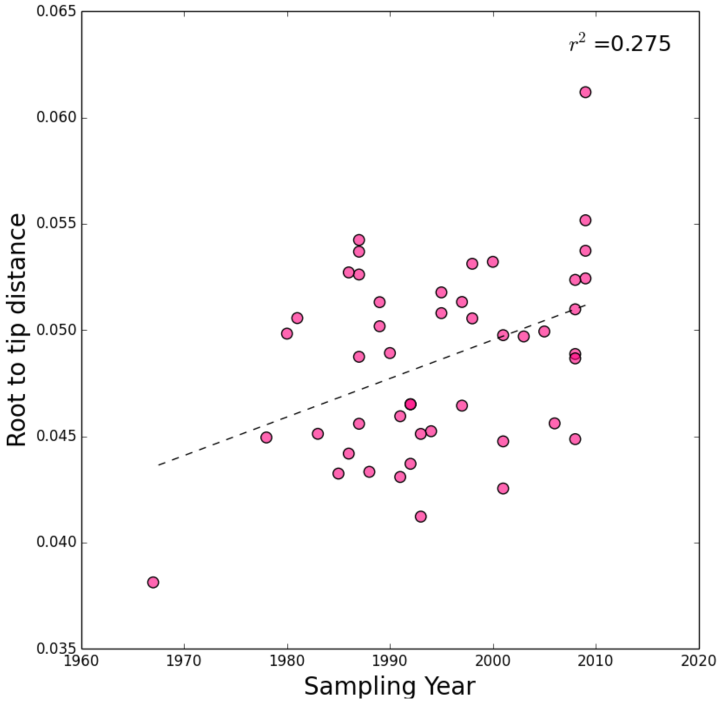

(B)

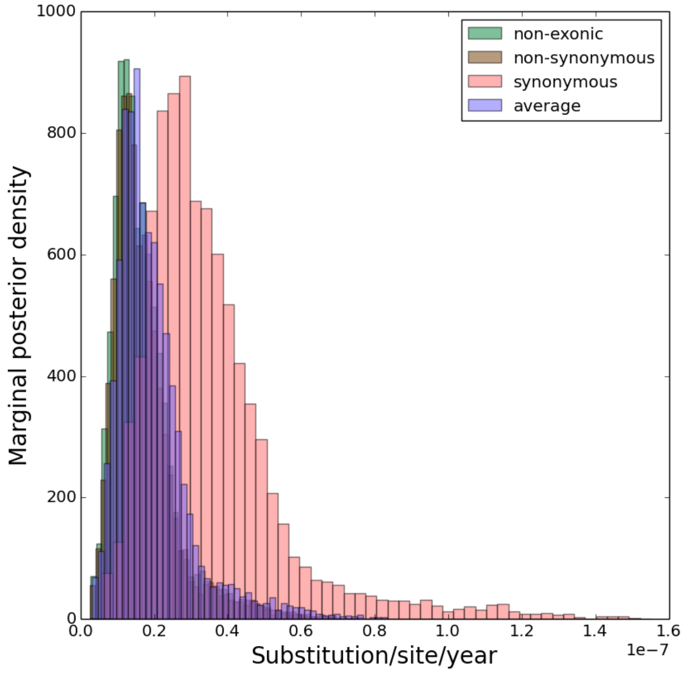

Figure S3. (A) Root-to-tip distances (mutations/site) estimated with Beast are correlated with collection date; (B) Marginal posterior densities of the substitution rates of the three data partitions (non-coding sites, nonsynonymous sites and synonymous sites), as estimated by tip-calibrated phylogenetic analysis.
