# Supplementary material for: Coexistence of Multiple Endemic and Pandemic Lineages of the Rice Blast Pathogen
Source: mBio. 2018 Apr 3;9(2):e01806-17. doi: 10.1128/mBio.01806-17 (PMC5885030; doi:10.1128/mBio.01806-17)
Supplement: FIG S4 [file mbo002183809sf4.docx]

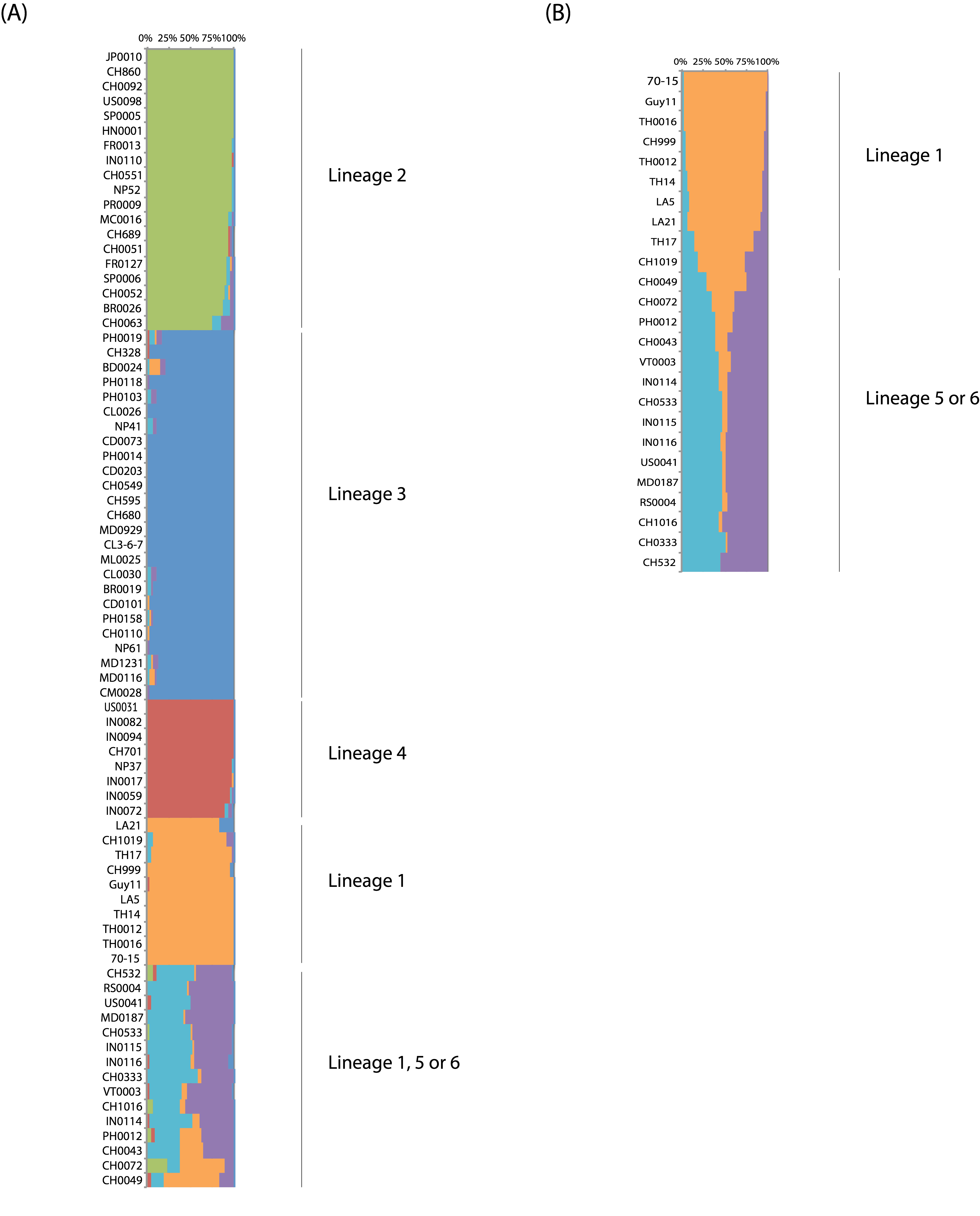


**Figure S4.** Proportions of ancestry in *K*=6 ancestral populations inferred with STRUCTURE program from 77 multilocus genotypes. Individuals were genotyped at 12 microsatellite loci and 164 SNP loci. Each individual is represented by a bar, partitioned into *K* segments representing the extent to which its genome is descended from each ancestral population.
